# Abnormal Organization of White Matter Network in Patients with No Dementia after Ischemic Stroke

**DOI:** 10.1371/journal.pone.0081388

**Published:** 2013-12-13

**Authors:** Lin Shi, Defeng Wang, Winnie C. W. Chu, Shangping Liu, Yunyun Xiong, Yilong Wang, Yongjun Wang, Lawrence K. S. Wong, Vincent C. T. Mok

**Affiliations:** 1 Department of Imaging and Interventional Radiology, The Chinese University of Hong Kong, Shatin, New Territories, Hong Kong, China; 2 Shenzhen Institutes of Advanced Technology, Chinese Academy of Sciences, Shenzhen, China; 3 CUHK Shenzhen Research Institute, Shenzhen, China; 4 Department of Medicine and Therapeutics, The Chinese University of Hong Kong, Shatin, New Territories, Hong Kong, China; 5 Department of Neurology, Beijing Tiantan Hospital, Capital Medical University, Beijing, China; Institute of Psychology, Chinese Academy of Sciences, China

## Abstract

Structural changes after ischemic stroke could affect information communication extensively in the brain network. It is likely that the defects in the white matter (WM) network play a key role in information interchange. In this study, we used graph theoretical analysis to examine potential organization alteration in the WM network architecture derived from diffusion tensor images from subjects with no dementia and experienced stroke in the past 5.4–14.8 months (N = 47, Mini-Mental Screening Examination, MMSE range 18–30), compared with a normal control group with 44 age and gender-matched healthy volunteers (MMSE range 26–30). Region-wise connectivity was derived from fiber connection density of 90 different cortical and subcortical parcellations across the whole brain. Both normal controls and patients with chronic stroke exhibited efficient small-world properties in their WM structural networks. Compared with normal controls, topological efficiency was basically unaltered in the patients with chronic stroke, as reflected by unchanged local and global clustering coefficient, characteristic path length, and regional efficiency. No significant difference in hub distribution was found between normal control and patient groups. Patients with chronic stroke, however, were found to have reduced betweenness centrality and predominantly located in the orbitofrontal cortex, whereas increased betweenness centrality and vulnerability were observed in parietal-occipital cortex. The National Institutes of Health Stroke Scale (NIHSS) score of patient is correlated with the betweenness centrality of right pallidum and local clustering coefficient of left superior occipital gyrus. Our findings suggest that patients with chronic stroke still exhibit efficient small-world organization and unaltered topological efficiency, with altered topology at orbitofrontal cortex and parietal-occipital cortex in the overall structural network. Findings from this study could help in understanding the mechanism of cognitive impairment and functional compensation occurred in patients with chronic stroke.

## Introduction

Ischemic stroke is one of the leading causes of adult disability, resulting from cessation of blood supply due to an occlusion of a cerebral artery. Patients suffered from ischemic stroke are at increased risk of developing vascular cognitive impairment [1], [2], which ranges in severity from mild and/or isolated cognitive impairment to vascular dementia. Vascular cognitive impairment with no dementia is a prodromal, mild stage of dementia, lying on a continuum between normal cognition and dementia [3]. Among survivors of ischemic stroke, the prevalence of post-stroke dementia is about 30%, and the risk of dementia is doubled as compared with subjects who have not had stroke [4]. Hence, identification of factors that cause cognitive impairment would benefit interventions to prevent the progression to dementia.

Multiple magnetic resonance imaging (MRI) techniques have been used to investigate the structural and functional changes in the brains of post-stroke patients. Structural MRI analyses included white matter (WM) lesion distribution [5], gray matter atrophy [6], and frontal lobe atrophy [7] were used to evaluate the cognitive impairment after ischemic stroke. Furthermore, resting-state functional MRI (rs-fMRI) was also used to identify the post-stroke connectivity changes [8]–[10]. Since diffusion tensor imaging is sensitive to detect the microstructural changes in WM [11], recent studies have investigated the post-stroke abnormalities of diffusion tensor measurements, such as mean diffusivity, fractional anisotropy (FA), and apparent diffusion coefficient [12]–[14]. In addition, complex network graphs that quantify interactions between brain regions have recently given new insights into the spontaneous reorganization of functional and structural brain networks after stroke [15]–[17]. Although WM damages have been found to be potentially associated with the development of cognitive impairment after ischemic stroke [14], [18], it remains unknown whether the overall WM organization of brain are affected in patients with no dementia after ischemic stroke.

Since diffusion MRI tractography is advantageous for investigating WM pathways and visualizing brain structural connectivity in vivo [19]–[21], graph theoretical approaches have been proposed to characterize the organization and architecture of the structural networks of the human brain [22]–[24]. Indeed, the alteration in topological organization of brain WM network have been demonstrated useful to understand the cognitive functions [25] and to find the characteristic mechanisms of psychiatric and neurological diseases [26]–[31]. These studies indicate that it is feasible to employ DTI tractography to investigate structural networks in patients with chronic stroke. Our goal in this study is to apply WM tractography and graph theoretic analysis to investigate abnormalities in the organization and architecture of the structural connectivity pattern at the macroscopic scale in patients after ischemic stroke, based on the following hypotheses: (i) Patients with chronic stroke could exhibit efficient small-world topology, as has been successfully established in several disease models including stroke model in rats [32]. On this basis we would like to further identify the possible alterations of overall topological properties compared with normal controls. Because either decreased small-world metrics related to impaired neural circuits directly caused by infarct lesions, or increased measurements reflecting excessive neuronal clustering and wiring after stroke [32] or as a compensation mechanism as described previously in schizophrenia [33], possibly occurs in chronic stroke patients; (ii) Regions previously considered to be responsible for a certain type of cognitive impairment (e.g., post-stroke depression [34], executive dysfunction [35]) could be identified with altered nodal characteristics, thus help to give an anatomical & topological manifestation of post-stroke functional abnormality; (iii) Correlations between Mini-Mental Screening Examination (MMSE) or National Institutes of Health Stroke Scale (NIHSS) scores and network characteristics can be possibly established. Since there are evidences suggest that functional or structural connectivity changes are correlated with MMSE score in amnestic mild cognitive impairment (aMCI) and Alzheimer's disease [36], and reflect the post-stroke motor network alterations [37] and severity of stroke in rat model [32].

## Materials and Methods

### Subjects

All the subjects were right-handed and were all informed and signed with a verbal and written consent form. Ethical approval was obtained from the Ethics Committee in the Chinese University of Hong Kong. Forty-seven patients with no dementia after ischemic stroke (29 males and 18 females; 68.7±9.6 years old) and 44 age- and gender-matched normal controls (NC) (21 males and 23 females; 68.8±8.3 years old), were recruited from the Prince of Wales Hospital in Hong Kong. Inclusion criteria for patients after ischemic stroke and no dementia were: (1) ischemic stroke within the past 5.4–14.8 months (average time 7.7±2.2 months), defined according to NINDS Stroke Data Bank criteria [38]; (2) age ≥50 years; (3) MMSE score ≥16; (4) competent to complete neuropsychological tests [39]; (5) verbal and written consent; (6) available to collect patients' medical history from their caregivers (spent at least three days per week nursing the patients in the last five years). Inclusion criteria for NCs were: (1) age ≥50 years; (2) competent to complete neuropsychological tests; please refer to the NINDS-CSN protocols in [39]; (3) verbal and written consent; (4) MMSE score ≥24. The patient and NC groups had median MMSE scores of 25 (range 18–30) and 29 (range 26–30), respectively. Exclusion criteria for both NC and patient group included history of central nervous system disorder or disease. Twenty patients with chronic stroke possibly had cognitive impairment (MMSE <24). No significant differences were found in age, gender and education between the two groups (all *p*>0.05).

### MRI Acquisition

All subjects were scanned using a clinical 3T MRI scanner with an 8-channel Sense head coil (Achieva, Philips Medical Systems) at the Prince of Wales Hospital in Hong Kong. For each subject, T1-weighted (T1W) images, fluid attenuation inversion recovery (FLAIR) images, and diffusion tensor images (DTI) covering the whole brain were acquired in the axial orientation. T1W images were obtained using 3D fast field echo imaging sequence with the following parameters: (repetition time [TR]  = 6.7 ms, echo time [TE]  = 1.6 ms, number of excitation [NEX]  = 2, field of view [FOV]  = 220 mm, flip angle  = 15°, matrix  = 256×256, slice  = 128, voxel size  = 0.86×0.86×1.4 mm^3^. FLAIR image was obtained using following parameters: TR  = 11000 ms, TE  = 200 ms, Inversion Time [TI]  = 2800 ms, NEX  = 1, FOV  = 220 mm, flip angle  = 90°, matrix  = 704×704, slice  = 58, voxel size  = 0.31×0.31×3 mm^3^. DTI was obtained using single-shot echo planar imaging sequence with the following parameters: 15 diffusion weighted volumes (b = 750 s/mm^2^), one non-diffusion-weighted volume (b = 0 s/mm^2^), TR  = 10900 ms, TE  = 84.5 ms, NEX  = 2, FOV  = 220 mm, flip angle  = 90°, matrix  = 256×256, slice  = 55, voxel size  = 0.86×0.86×3 mm^3^.

### Delineation and Mapping of Infarcts

**Figure 1:**
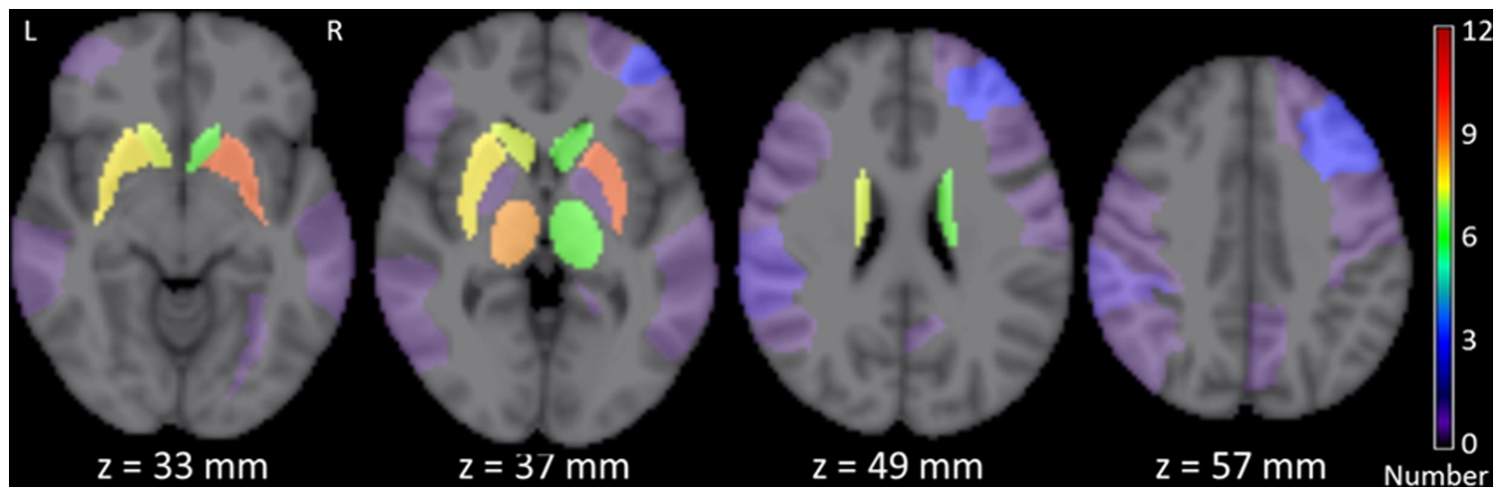
Distribution of infarct location of whole post-stroke group overlaid on 4 different slices in the ICBM152 template in MNI space. The number of infarcts was color-coded and superimposed on the ICBM152 template.

The FLAIR image of each subject with chronic stroke was first co-registered to T1W images using FMRIB's Linear Registration Tool (FLIRT) [40]. The infarcts of each patient were manually delineated on FLAIR images with T1W image loaded as a reference by an experienced neuroradiologist (YYX). Then T1W image was co-registered to the ICBM152 template in Montreal Neurological Institute (MNI) space using FMRIB's Non-linear Image Registration Tool (FNIRT) [41], and the delineated infarcts was transformed to MNI space. The infarcts were masked out during the registration by setting their voxel values in the weighting volume as zero. Then the infarcts on gray matter and white matter were mapped to automated anatomical labeling (AAL) [42] and ICBM DTI-81 white matter labels atlas [43] respectively. The location of infarcts for each subject was determined by AAL and DTI-81 atlas with visual quality control, and the spatial distribution of infarcts of whole patient group was obtained (Table 1 and Figure 1).

**Table 1:** The number of stoke patients with infarcts within different brain regions.

Location of infarct		# Patients
Frontal	Left side only	7
	Right side only	3
	Bilateral	4
Parietal-occipital	Left side only	2
	Right side only	1
	Bilateral	1
Temporal	Left side only	0
	Right side only	0
	Bilateral	1
Basal ganglia	Left side only	3
	Right side only	7
	Bilateral	6
Thalamus	Left side only	3
	Right side only	6
	Bilateral	1
Infratentorial		15
Infarct Volume (mm^3^) (median (IQR))		169.5 (648.0)

### Connectivity Network Construction

#### Image Preprocessing

**Figure 2:**
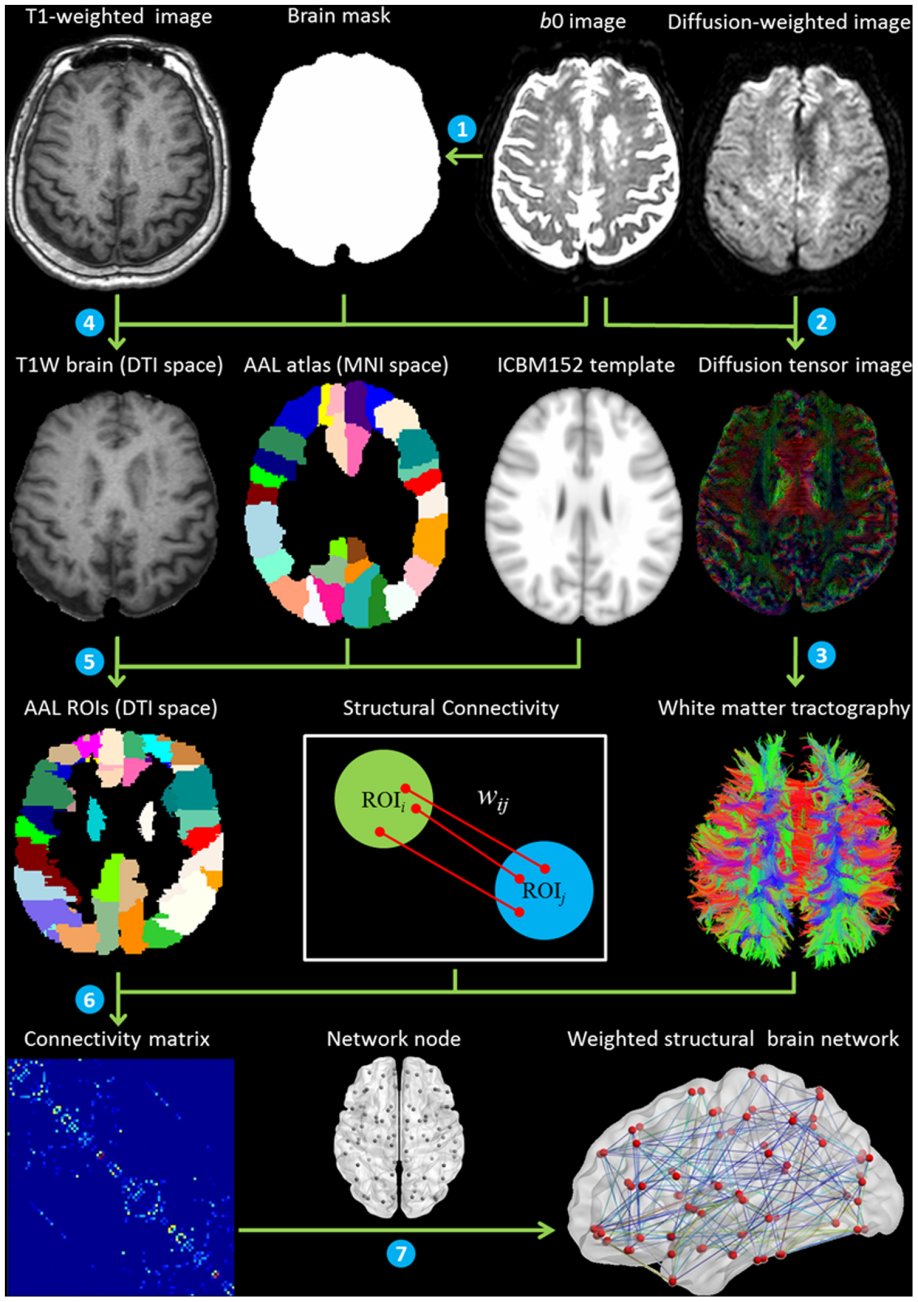
Whole schematic flowchart of WM network construction, (1) brain extraction, (2) DTI preprocessing and diffusion tensor model estimation, (3) white matter fiber tracking, (4) co-registration T1W image with non-diffusion-weighted *b*0 image, (5) mapping T1W image to ICBM152 template and warping the AAL atlas to native DTI space, (6) structural connectivity mapping and connectivity matrix generation, (7) weighted WM network construction.

The whole schematic flowchart of WM connectivity network construction is shown in Figure 2. Artifacts induced by head motion and eddy current in DTI data were processed by affinely registering diffusion-weighted volumes to the non-diffusion-weighted *b*0 volume using the FMRIB's Diffusion Toolbox of FSL [44]. Skull and non-brain tissue were removed in the masked and corrected diffusion data by applying the FSL Brain Extraction Tool [45] to *b*0 image. The T1W image of each individual subject was co-registered to their *b*0 image and transformed to native DTI space using FLIRT.

#### White Matter Tractography

For each individual subject, diffusion tensor model was first estimated using the linear least-squares fitting method [46]. White matter tracts of the whole brain were subsequently reconstructed using the fiber assignment by continuous tracking (FACT) algorithm [47] with the FA threshold at 0.15 and tracking turning angular threshold at 35° between two connections. Afterwards, a spline filtering was applied to smooth the streamline tractography. Diffusion Toolkit 0.6 (http://www.nitrc.org/projects/trackvis/) was used for DTI data processing and tractography in this work.

#### Brain Parcellation

The brain was automatically partitioned into 90 non-cerebellar regions (45 cortical and subcortical regions in each hemisphere, Table 2) using the AAL parcellation atlas. For each subject, individual T1W image in the native DTI space was mapped to the ICBM152 template in MNI space using FNIRT, and the AAL atlas was inversely warped to the native DTI space by applying nearest-neighbor interpolation. The regions with detected infarcts were masked out before performing image registration, so that image registration was based on reliable information from image of normal tissues.

**Table 2:** Brain regions in the AAL atlas.

*AAL index (left, right)*	*Regions*	*Abbr.*	*AAL index (left, right)*	*Regions*	*Abbr.*
2001, 2002	Precentral gyrus	PreCG	5021, 5022	Lingual gyrus	LING
2101, 2102	Superior frontal gyrus (dorsolateral)	SFGdor	5101, 5102	Superior occipital gyrus	SOG
2111, 2112	Superior frontal gyrus (orbital)	ORBsup	5201, 5202	Middle occipital gyrus	MOG
2201, 2202	Middle frontal gyrus	MFG	5301, 5302	Inferior occipital gyrus	IOG
2211, 2212	Middle frontal gyrus (orbital)	ORBmid	5401, 5402	Fusiform gyrus	FFG
2301, 2302	Inferior frontal gyrus (opercular)	IFGoperc	6001, 6002	Postcentral gyrus	PoCG
2311, 2312	Inferior frontal gyrus (triangular)	IFGtriang	6101, 6102	Superior parietal gyrus	SPG
2321, 2322	Inferior frontal gyrus (orbital)	ORBinf	6201, 6202	Inferior parietal gyrus	IPG
2331, 1232	Rolandic operculum	ROL	6211, 6212	Supramarginal gyrus	SMG
2401, 2402	Supplementary motor area	SMA	6221, 6222	Angular gyrus	ANG
2501, 2502	Olfactroy cortex	OLF	6301, 6302	Precuneus	PCUN
2601, 2602	Superior frontal gyrus (medial)	SFGmed	6401, 6402	Paracentral lobule	PCL
2611, 2612	Superior frontal gyrus (medial orbital)	ORBsupmed	7001, 7002	Caudate	CAU
2701, 2702	Rectus gyrus	REC	7011, 7012	Putamen	PUT
3001, 3002	Insula	INS	7021, 7022	Pallidum	PAL
4001, 4002	Anterior cingulate gyrus	ACG	7101, 7102	Thalamus	THA
4011, 4012	Median cingulate gyrus	MCG	8101, 8102	Heschl gyrus	HES
4021, 4021	Posterior cingulate gyrus	PCG	8111, 8112	Superior temporal gyrus	STG
4101, 4102	Hippocampus	HIP	8121, 8122	Temporal pole (superior)	TPOsup
4111, 4112	Parahippocampal gyrus	PHG	8201, 8202	Middle temporal gyrus	MTG
4201, 4202	Amygdala	AMYG	8211, 8212	Temporal pole (middle)	TPOmid
5001, 5002	Calcarine cortex	CAL	8301, 8302	Inferior temporal gyrus	ITG
5011, 5012	Cuneus	CUN	-	-	-

#### Structural Connectivity Mapping

In the native DTI space, two AAL regions were considered to be connected if at least one fiber was presented between them [19]. The fiber connection density between two AAL regions was defined as the normalized number of fibers per unit volume [23], [48], [49]. The number and length of fibers connecting each pair of AAL regions were used to calculate the fiber connection density,
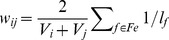
(1)where *V_i_* and *V_j_* denote the volume of AAL regions *i* and *j*, respectively, *F_e_* denotes the set of fibers connecting regions *i* and *j*, and *l_f_* denotes the length of fiber *f* along its trajectory. Taking fiber connection density between two AAL regions as its connection weight, a weighted 90×90 structural connectivity matrix for each subject was generated. Each node of the constructed network corresponded to one AAL region.

### Network Graph Analysis

The topological organization of the weighted structural connectivity network was characterized at the nodal and global levels using the Brain Connectivity Toolbox [50] based on graph theory. The nodal behavior was quantified in terms of connection strength, local clustering coefficient, betweenness centrality, regional efficiency and vulnerability. The global network architecture was described in terms of the global connection strength, global clustering coefficient, characteristic path length, global betweenness centrality, global vulnerability, normalized clustering coefficient, normalized path length, and small-worldness. Each metric provided a different viewpoint to describe major features of the large-scale architecture.

#### Regional Nodal Characteristics

The connection strength 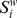 of each node *i* in the weighted network was computed as the sum of the weights of all its connections, which provided information on the total level of connectivity [30], [50],
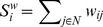
(2)where *w_ij_* is the weight between nodes *i* and *j* in the network, and *N* is the set of all nodes in the network.

The local clustering coefficient 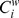 of each node *i* in the weighted network was defined as the likelihood that its neighbors were interconnected to each other, which described the strength of one node and its neighbors were clustered [51], [52],
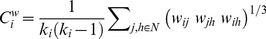
(3)where *k_i_* is the node degree defined as the number of connections to node *i*. The local clustering coefficient of the nodes with less than two connections was set as zero.

The betweenness centrality 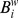 of each node *i* in the weighted network was defined as the fraction of all shortest paths in the network that pass through it, which was essentially a measurement of the influence of a node over the information flow between itself and other nodes [53], [54],
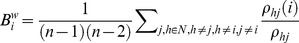
(4)where 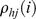 is the number of shortest path between nodes *h* and *j* that passed through node *i*, *n* is the total number of nodes.

The regional efficiency 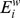 of each node *i* in the weighted network was defined as the inverse of harmonic mean of the shortest path length between itself and all other nodes, which was used to quantify the importance of a node in the communication within the network [55],
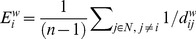
(5)where 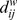 is the length of the shortest weighted path between nodes *i* and *j* in the weighted network,
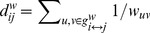
(6)where 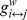 indicates the shortest weighted path between nodes *i* and *j*.

The vulnerability 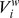 of each node *i* in the weighted network is defined as the drop in global efficiency when this node and its connections are removed from the network graph, which was used to identify the most indispensable node to network efficiency [24], [56],
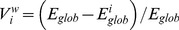
(7)where 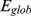 is the global efficiency of the network, 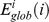 is the global efficiency of the network after removing node *i*. The global efficiency is defined as the inverse of the harmonic mean of the shortest path length between every two nodes in a network,
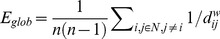
(8)


#### Overall Graph characteristics

The global strength 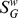 of the weighted network was computed as the mean connection strength across all nodes in the network,
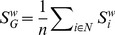
(9)


The global clustering coefficient 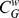 was defined as the average of the local clustering coefficient 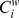 over all nodes in the network [52],
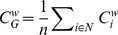
(10)


The weighted characteristic path length 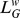 of the entire network was measured by the harmonic mean of the shortest path between every two nodes and was equivalent to the inverse of the global efficiency, which expressed how well the overall network was connected and represents the capacity to exchange information [57], [58],
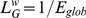
(11)


The global betweenness centrality 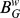 was calculated as the average of the values of the nodal betweenness 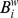 over all nodes,
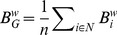
(12)


The global vulnerability 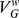 of the entire network was defined as the maximum vulnerability for all of its nodes [56],
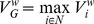
(13)


The measure of small-worldness 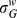 was defined as ratio between the normalized clustering coefficient 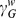 and the normalized characteristic path length 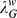, which denoted the small-world organization of the weighted network [59].
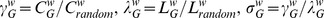
(14)where the 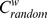 and 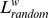 are the average of global clustering coefficient and characteristic path length of 100 matched random networks that preserved the same number of nodes, edges, and degree distribution as the real network.

#### Hub Identification

The putative hubs were usually the nodes with high connection strength, high betweenness centrality, high regional efficiency, low clustering coefficient, and/or high vulnerability, which played a crucial role in fast information transferring and efficient integration of information in whole network communication [22], [30], [60]–[62]. The hubs of the structural brain network were identified using the hub score, a rank-based analysis of the multiple mean nodal metrics. The hub identification criteria included (1) within the top 20% nodes showing the highest mean strength 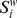; (2) within the top 20% nodes showing the lowest local clustering coefficient 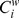; (3) within the top 20% nodes showing the highest betweenness centrality 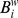; (4) within the top 20% nodes showing the highest regional efficiency 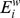; (5) within the top 20% nodes showing the highest global vulnerability 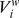. For each node, a hub score between 0 to 5 was assigned. Nodes with a hub score of 2 or higher were considered as hubs. [22], [61].

### Statistical Analysis

#### Overall Graph Characteristics

Differences in overall graph characteristics (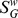, 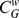, 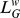, 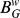, 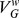, 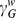, 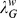, and 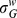) between the patient and NC groups were examined using permutation testing [30], [31], [60]. First, the observed values of the test were calculated as the differences in the measured overall graph characteristics between the two groups. Then the subjects in both groups were pooled and randomly assigned to either one of two groups consisting of the same size as the original patient and NC groups. The differences in overall graph characteristics between the two random groups were computed. This procedure was repeated for 5,000 times. The one-tailed *p-*value was then calculated as the proportion of sampled permutations where differences that were greater than (or smaller than) the observed values. A significance threshold of *p* = 0.05 (uncorrected) was used for testing the overall graph characteristics.

#### Regional Nodal Characteristics

The statistical analysis for the regional nodal characteristics was similar to those for global network characteristics. The regional characteristics (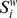, 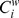, 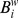, 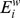, and 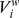) of each node in the network were compared between the patient and NC groups for all nodes using the above-mentioned permutation test. The one-tailed *p*-value was calculated and the false discovery rate (FDR) [63] with *q* = 0.05 was used to correct the multiple comparisons. The FDR was the expected proportion of false positives among significant results.

#### Correlation with the MMSE and NIHSS score

The Pearson partial correlation between the MMSE score and the network properties, and between the NIHSS score and the properties at both the global and nodal levels were evaluated in patient group while controlling for age and gender. The significance level for testing the overall graph characteristics was set at *p*<0.001 (uncorrected). A statistical significance level of *p*<0.001 (uncorrected) was used for testing the regional nodal characteristics.

## Results

### Overall Topological Properties

**Figure 3:**
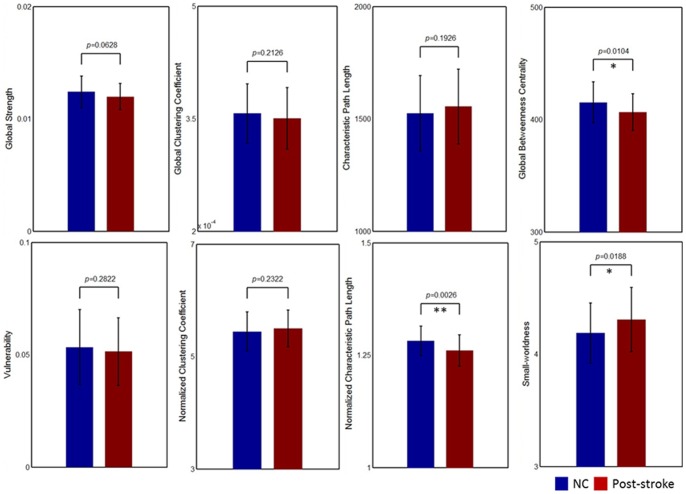
Group comparison of the overall graph characteristics, i.e., connection strength (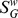), global clustering coefficient (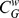), characteristic path length (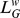), global betweenness centrality (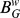), global vulnerability (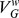), normalized clustering coefficient (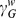), normalized path length (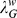), and small-worldness (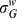). Single asterisk (*) represents a significant difference level at *p*<0.05, while a double asterisk (**) represents a significant difference level at *p*<0.01.

Both normal controls and patients with chronic stroke exhibited efficient small-world topology (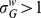) in structural networks (NC: 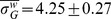, patients: 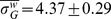). In addition, a significant increase of small-worldness (*p* = 0.0226) and a decrease of normalized characteristic path length (*p* = 0.0026) in patient group were observed (Figure 3). Furthermore, as compared with the overall graph characteristics of patients, no significant differences in global strength 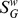 global clustering coefficient 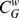, characteristic path length 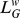, and global vulnerability 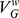 (all *p*>0.05) were found (Figure 3). The global betweenness centrality 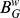 (*p* = 0.0104) was significantly decreased in patient group (Figure 3).

### Distribution of Hub Region

**Figure 4:**
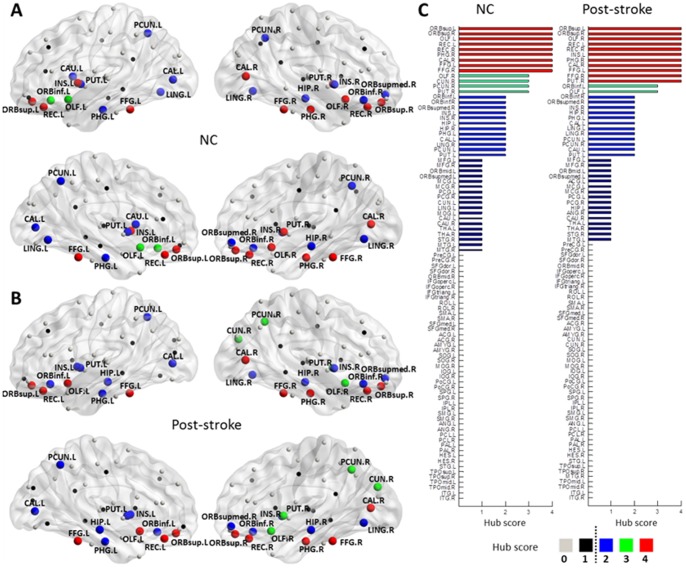
Hub distributions and node-specific hub score of WM networks in both NC and post-stroke groups. (A) Hubs in the NC group are distributed in 9 orbitofrontal regions, 2 temporal regions, 2 parietal regions, 4 occipital regions, 3 limbic regions, 3 basal ganglia regions, and bilateral insula. (B) Hubs in the post-stroke group are distributed in 9 orbitofrontal regions, 2 temporal regions, 2 parietal regions, 4 occipital regions, 4 limbic regions, 2 basal ganglia regions, and bilateral insula. (C) Descendingly sorted 90 AAL brain regions using hub scores for both NC and post-stroke groups.

In both NC and patient groups, 25 hubs were identified by multiple nodal metrics (Table 3, Figure 4). In particular, 23 of them were identified in both groups e.g. bilateral orbital part of superior frontal gyrus [ORBsup], bilateral olfactory cortex [OLF], bilateral rectus gyrus [REC], bilateral parahippocampal gyrus [PHG], bilateral calcarine cortex [CAL], bilateral fusiform gyrus [FFG], bilateral precuneus [PCUN], bilateral orbital part of the inferior frontal gyrus [ORBinf], right medial orbital part of superior frontal gyrus [ORBsupmed.R], bilateral insula [INS], right hippocampus [HIP.R], right lingual gyrus [LING.R], and bilateral putamen [PUT]. For hub scores between post-stoke to NC, *hub (hub score post-stoke→hub score NC):* OLF.L (3→4) and PCUN.R (2→3) were increased. OLF.R (4→3), PUT.R (4→3), ORBinf.L (3→2), and INS.L (4→2) were decreased. Two regions, the left caudate [CAU.L] (2→1) and left lingual gyrus [LING.L] (2→1), were identified as hubs in the patient group but not in the NC group. Two regions, left hippocampus [HIP.L] (1→2) and right cuneus [CUN.R] (0→3), were identified as hubs in the NC group but not in the patient group.

**Table 3:** Hubs and hub scores in NC and post stroke patients.

NC		Post-stroke	
Hubs	Hub score	Hubs	Hub score
ORBsup L	4	ORBsup L	4
ORBsup R	4	ORBsup R	4
OLF L	4	OLF R	4
REC L	4	REC L	4
REC R	4	REC R	4
PHG R	4	INS L	4
CAL R	4	PHG R	4
FFG L	4	CAL R	4
FFG R	4	FFG L	4
OLF R	3	FFG R	4
CUN R	3	PUT R	4
PCUN R	3	ORBinf L	3
PUT R	3	OLF L	3
ORBinf L	2	ORBint R	2
ORBinf R	2	ORBsupmed R	2
ORBsupmed.R	2	INS R	2
INS L	2	HIP R	2
INS R	2	PHG L	2
HIP L	2	CAL L	2
HIP R	2	LING L	2
PHG L	2	LING R	2
CAL L	2	PCUN L	2
LING R	2	PCUN R	2
PCUN L	2	CAU L	2
PUT L	2	PUT L	2

### Nodal Characteristics Alteration

**Figure 5:**
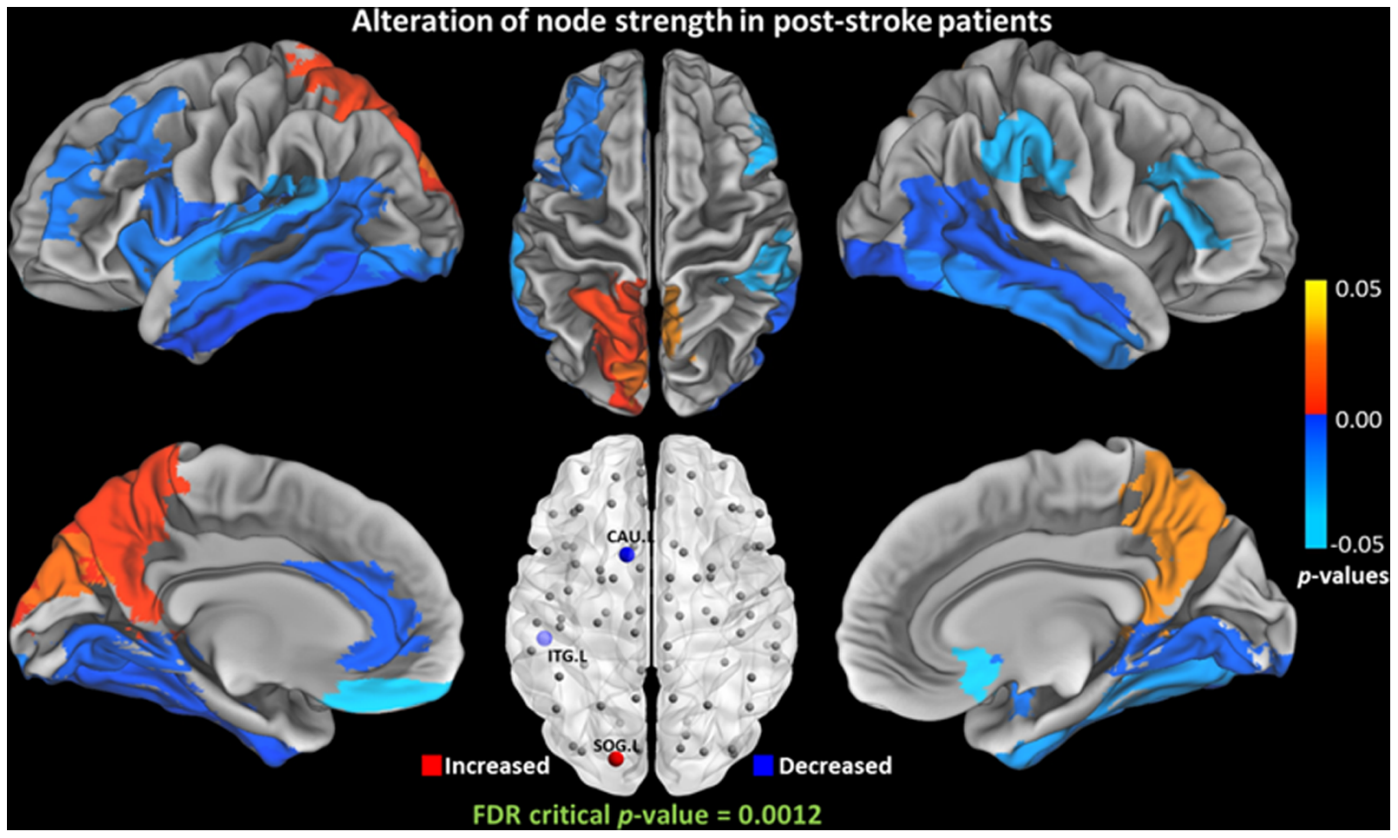
Group differences of node-specific connection strength (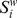) between NC and post-stroke groups. Significant increase was observed in left superior occipital gyrus [SOG.L]. Significant decrease was observed in left caudate [CAU.L] and left Inferior temporal gyrus [ITG.L].

**Figure 6:**
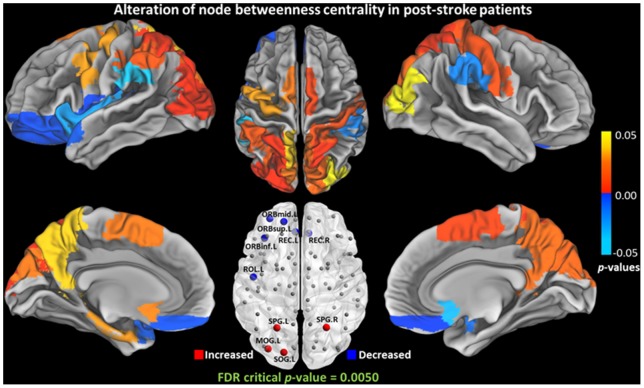
Group differences of nodal betweenness centrality (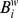) between NC and post-stroke groups. Significant increase was observed in left superior parietal gyrus [SPG.L], right superior parietal gyrus [SPG.R], left superior occipital gyrus [SOG.L], and left middle occipital gyrus [MOG.L]. Significant decrease was observed in left orbital part of superior frontal gyrus [ORBsup.L], orbital part of middle frontal gyrus [ORBmid.L], orbital part of inferior frontal gyrus [ORBinf.L], left gyrus rectus [REC.L], right gyrus rectus [REC.R], and left rolandic operculum [ROL.L].

**Figure 7:**
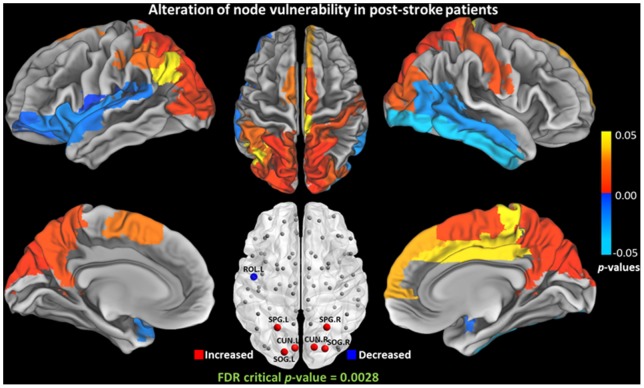
Group differences of nodal vulnerability (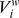) between NC and post-stroke groups. Significant increase was observed in left superior parietal gyrus [SPG.L], and right superior parietal gyrus [SPG.R], left cuneus [CUN.L], right cuneus [CUN.R], left superior occipital gyrus [SOG.L], and right superior occipital gyrus [SOG.R]. Significant decrease was observed in left rolandic operculum [ROL.L].

The nodal characteristics (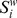, 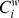, 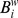, 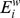, and 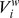) of each cortical and subcortical region between the two groups were further compared. The 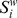, 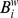, and 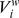 of a number of regions were altered (Table 4, Figures 5, 6, and 7). No significant alteration of the local clustering coefficient 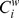 and regional efficiency 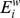 (FDR critical *p*-value  = 0) were found between the two groups. The 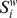, 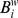, 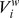 of left superior occipital gyrus [SOG.L] were significantly increased.

**Table 4:** Nodal characteristics comparison between post-stroke and NC.

Post-stroke group compared to NC	Increased	Decreased
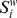 (Figure 5)	SOG. L (*p* = 0.0002★)	CAU. L (*p* = 0.0006★)
		ITG. L (*p* = 0.0012★)
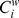	No significant alteration	No significant alteration
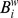 (Figure 6)	SPG. L (*p* = 0.0016★)	ORBsup. L (*p* = 0.0038★)
	SPG. R (*p* = 0.0022★)	ORBmid. L (*p* = 0.0028★)
	SOG. L (*p* = 0.0004★)	ORBinf. L (*p* = 0.0050★)
	MOG. L (*p* = 0.0010★)	REC. L (*p* = 0.0048★)
		REC. R (*p* = 0.0004★)
		ROL. L (*p* = 0.0024★)
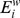	No significant alteration	No significant alteration
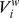 (Figure 7)	SPG. L (*p* = 0.0004★)	ROL. L (*p* = 0.0008★)
	SPG. R (*p* = 0.0008★)	
	CUN. L (*p* = 0.0028★)	
	CUN. R (*p* = 0.0014★)	
	SOG. L (*p* = 0.0002★)	
	SOG. R (*p* = 0.0024★)	

indicated the region survived critical FDR threshold.

### Correlation with MMSE and NIHSS scores

No significant correlations were found between MMSE score and the network properties in patient group at both the global and nodal levels. The NIHSS score of patient is correlated with the betweenness centrality of right pallidum [PAL.R] (*p* = 0.0000, *r* = 0.644) and the local clustering coefficient of left superior occipital gyrus [SOG.L] (*p* = 0.0002, *r* = 0.5523). No significant correlation was found between NIHSSS and overall graph characteristics.

## Discussion

### Small-world Organization

Both normal controls and patients with chronic stroke were found to demonstrate small-world architectures. In particular, patients with chronic stroke displayed small-worldness close to the values of the structural networks in previous DTI-based studies with similar age groups [25]–[27]. Although WM networks of patients with chronic stroke showed prominent small-world attributes, several small-world measurements were found to be significantly altered. The patients showed increased small-worldness and decreased normalized characteristic path length, suggesting a more regular organization of WM network in patients with chronic stroke. Since there are no difference in characteristic path length (global level) and regional efficiency (nodal level), the decreased normalized characteristic path length is not necessarily an advantage in a complex network. The effective balance between local specialization and global integration arising from the small-world nature of anatomical connectivity possibly conserved in patients with chronic stroke. The increased small-worldness has also been observed in schizophrenia [33], during recovery from traumatic brain injury [64], and during recovery from stroke [32]. Furthermore, as patients with chronic stroke have not shown strong changes in local and global clustering coefficient, characteristic path length, and regional efficiency, our findings suggest the topological efficiency is not unaltered between patients and NCs.

### Alteration of Hub Distribution

Compared with the hub criteria based on the level of single nodal metrics in previous DTI-based studies, such as the betweenness centrality [65], [66] and regional efficiency [26], [28], the rank-based analysis of multiple nodal characteristics are more balanced and comprehensive. Thus, the identified hubs are partly consistent with those obtained from previous similar studies [23], [30], [31], [67]. Our results indicate no significant difference of the hub distribution between groups of NC and patient with chronic stroke (NC/post-stroke: 9/9 orbitofrontal regions, 2/2 temporal regions, 2/2 parietal regions, 4/4 occipital regions, 3/4 limbic regions, 3/2 basal ganglia regions, and bilateral insula, 12/11 left hemispherical regions, 13/14 right hemispherical regions) (Figure. 3). Increased central role of right parietal-occipital cortex and decreased central role of left parietal-occipital cortex are observed in patients with chronic stroke, supported by the fact that total infarct number in left hemisphere is greater than that in right hemisphere (Figure. 1), since no difference in lesion volume (*p* = 0.301) or course of stroke (*p* = 0.864) was found between the patients with left and right lateralized infarct lesions.

### Altered Nodal Characteristics

Group differences in betweenness centrality reflect the effects of the disease on the global roles of every node region. Reduced betweenness centrality is mainly observed in orbitofrontal cortex. The finding of decreased centrality in frontal lobe is in line with the pattern of frontal lobe atrophy in ischemic stroke patients [7], [68]. The orbitofrontal cortex is a prefrontal cortex region in the frontal lobes which is involved in the cognitive processing of decision-making. Five regions with decreased centrality were found in left orbitofrontal cortex of patients after ischemic stroke, supported by the fact that patients with subcortical ischemic vascular disease often have executive dysfunction [35]. Furthermore, cortical thinning in orbitofrontal gyrus has shown in vascular cognitive impairment associated with small vessel ischemic disease [69]. In this study, 20/47 patients exhibited cognitive impairment which support the findings of the orbitofrontal abnormalities. Decreased centrality is observed in four orbitofrontal hub regions, suggesting a decreased hub role of orbitofrontal regions in the brain network of patients. Interestingly, increased betweenness centrality is observed in parietal-occipital cortex. Increased centrality may indicate a shift to more parietal-occipital brain regions as a possible compensatory mechanism for decreased centrality of orbitofrontal regions. As the total infarct number in the left hemisphere is greater than that in the right hemisphere, the locations with altered centrality between the left and right hemispheres have showed the most asymmetry.

Apart from betweenness centrality, vulnerability is also used to characterize the influence of nodes in a network. As betweenness and vulnerability quantify the node in different aspects, the nodes with higher betweenness are not necessarily more vulnerable [19]. From the results in this study, the increased vulnerability is consistent with the increased betweenness in parietal-occipital cortex. Nevertheless, a decreased betweenness is found in the prefrontal cortex but no reduced vulnerability is observed. The occipital regions with increased vulnerability include cuneus [CUN] and superior occipital gyrus [SOG] in bilateral occipital lobe, which are important for visual processing. These changes suggest that these regions have increased influence in the brain communication compared with NCs. This could be a potential evidence for understanding of the visual system recovery in patients with chronic stroke. Particularly, we have observed reduction in both centrality and vulnerability in left rolandic operculum [ROL.L], which is a key component for speech processing [70]. This finding is supported by the finding reported in the reduced white matter integrity and damaged tracts related to speech processing in post-stroke patients [71]. The structural abnormality of this area was also reported to be associated with stuttering [72].

The connection strength is the most fundamental network measure, and provides information of the total degree of connectivity. Decreased connection strength is found in the left caudate [CAU.L] and left inferior temporal gyrus [ITG.L]. The altered connection strength in CAU.L is consistent with the pattern of altered communicability [16]. The region of ITG.L is known to be included in the default mode network [73]. The rs-fMRI has revealed the association between altered functional connectivity of left middle temporal cortex in the DMN and post-stroke depression severity [34]. The decreased structural connectivity may give an anatomical comprehension of post-stroke depression. Imaging evidence also reveals the connectivity alteration of this region in post-stroke aphasia recovery [74], [75]. Specifically, the left superior occipital gyrus [SOG.L] presents significant increase in connection strength, centrality, and vulnerability, which is supported by reported gray matter atrophy in patients with ischemic stroke [6]. Recent research has associated degraded oral reading performance with acute left hemispheric stroke [76]. The betweenness of SOG.L is also larger than its counterpart in the right hemisphere in normal subjects [19].

### Correlation with the MMSE and NIHSS scores

Significant correlations were failed to be established between the MMSE score and the global or nodal characteristics. On one hand, the subjects studied are patients with no dementia, which means only mild cognitive impairment could be identified. On the other hand, MMSE provides only a general evaluation of cognitive function and suggests a compromised sensitivity in detecting mild cognitive dysfunction [77]. Furthermore, the severity of sensorimotor dysfunction, which is the major deficit after ischemic stroke, can not be reflected by MMSE. By contrast, NIHSS with more emphasis put on the aspects of sensorimotor functions was found to be correlated with the betweenness centrality of right pallidum [PAL.R] and the local clustering coefficient of SOG.L. Pallidum is mainly involved in the regulation of voluntary movement, and its change is supported by previous structural studies which revealed increased grey matter volume and cortical thickness in a series of motor-related areas during recovery from stroke [78], [79]. Besides, the correlation between clustering coefficient of SOG.L and NIHSS further confirmed the increased connection strength, centrality, and vulnerability observed in this region, suggesting its increasingly critical role in the reorganized network after stroke.

### Methodological Issues

Some methodological issues should be taken into account when interpreting our results. First, we used AAL atlas and FNIRT in FSL to perform brain parcellation. Since there is no widely accepted standard to construct cortical and subcortical regions in the brain, network nodes defined using atlas mapping are more valuable than random parcellation. Using different atlases, i.e., AAL atlas (90 cortical and subcortical regions), the Harvard–Oxford atlas (110 cortical and subcortical regions), Destrieux cortical atlas (66 cortical regions) [80], and LONI Probabilistic Brain Atlas (54 cortical regions) [81], different node sets would be generated and thus different properties of network would be produced. Although a research has suggested that the properties of WM network are highly conserved over multiple atlases and spatial resolution [82], different parcellation strategies affect WM connectivity and may result in different topological properties [83]. Moreover, voxel level (small area level) partition of cerebral cortex may be finer than atlas level partition if the parcellation methods are precise.

Second, we used fiber connection density as the inter-regional WM connectivity in the construction of the graphs. Previous researches have suggested different connectivity matrices for the WM network construction, i.e., fiber connection density [23], fiber number (FN) [28], mean FA [25], normalized fiber number [82], [84], and multiplication between FN and mean FA [27]). FN-based connectivity matrices are used to describe the interconnection of fiber tracts among regions of interest and should always be normalized, as the number of fibers detected varies among individuals. Mean FA is computed by averaging FA values along the tract and is used to describe the organization of the underlying white matter. Although the physiological meaning of these measures is poorly understood, both FN and FA based connectivity matrices are commonly used to characterize white matter integrity, and similar results have been found from FN-weighted and FA-weighted network [27], [31]. Compared with FN and FA, fiber connection density involved geometric measures including the volume of the cortical regions and the mean path length of fibers connecting each pair of regions is more stable.

Third, we used rank-based analysis of multiple nodal characteristics to identify the hub regions, and used hub score to describe the central role of hubs. Since there is no gold standard to define anatomical hubs, single or multiple nodal measures were usually used in other DTI-based studies [28], [30], [31], [49]. Compared with the level of single nodal metric, the rank-based criterion based on multiple nodal characteristics is more comprehensive to define a hub, and the hub score gives a quantitative description of its central role in the network.

## Conclusions

We have applied DTI tractography, WM connection mapping, and graph theoretic analysis to demonstrate altered organization of WM network in patients with chronic stroke. In this study, we comprehensively investigated different characteristics of WM connection in patients with chronic stroke and our findings suggest that patients have reduced betweenness centrality at orbitofrontal cortex, and increased betweenness centrality and vulnerability at parietal-occipital cortex, and exhibit efficient small-world organization and unaltered topological efficiencies. Compared to the NC, patients with chronic stroke exhibit significant increase of small-worldness, and connection strength 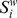, betweenness centrality 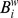, and vulnerability 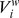 at left superior occipital gyrus [SOG.L], but significant decrease of normalized characteristic path length 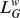 and global betweenness centrality 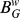. Our results also demonstrated the MMSE score of patients with chronic stroke is not significantly correlated with network properties.

The abnormities at orbitofrontal cortex and parietal-occipital cortex may affect the information exchange, but need further verification by fMRI studies to understand the functional difference between groups. The results from this study provide extra information for understanding of white matter connectivity changes in patients with chronic stroke in addition to the diffusion parameters obtained in the previous studies [85]–[87].
